# Saving the critically injured trauma patient: a retrospective analysis of 1000 uses of intraosseous access

**DOI:** 10.1186/1757-7241-23-S2-A15

**Published:** 2015-09-11

**Authors:** Philippa H Lewis, Chris R Wright

**Affiliations:** 1 Royal Army Medical Corps, 4 Armoured Medical Regiment, Aldershot, UK; 2 Emergency Department, Imperial College NHS Foundation Trust, London, UK

## Objective

Intraosseous access (IO) is becoming increasingly accepted in adult populations as an alternative to peripheral vascular access; however there is still insufficient evidence in large patient groups supporting its use.

## Methods

Retrospective review. This paper reports on the use of IO devices over a 7 year period from August 2006 to August 2013 during combat operations in Afghanistan. A database search of the Joint Theatre Trauma Registry (JTTR) was carried out looking for all the incidences of IO access use during this time. Excel^TM^ (Microsoft) was used to manage the dataset and perform descriptive statistics on the patient demographics, injuries, treatments and complications that were retrieved.

## Results

1014 IO devices were used in 830 adult patients with no major complications. The rate of minor complications, the majority of which were device failure, was 1.38%. 5124 separate infusions of blood products or fluids occurred via IO access, with 36% being of packed red cells. On average each casualty received 6.95 different infusions of blood products and fluids, and 3.28 separate infusions of drugs through IO access. 32 different drugs were infused to 367 patients via IO, the most frequent being anaesthetic agents. IO access was used in the pre-hospital environment, during tactical helicopter evacuation and within hospitals.

## Conclusion

IO access can be used to administer a wide variety of life saving medications quickly, easily and with low complication rates. This highlights its valuable role as an alternative method of obtaining vascular access, vital when resuscitating the critically injured trauma patient.

